# Non-Stoichiometric Effects on Viscoelasticity in DGEBA-EDA Systems: Insights from Brillouin Light Scattering

**DOI:** 10.1021/acs.jpcb.4c06661

**Published:** 2024-10-25

**Authors:** Mikolaj Pochylski

**Affiliations:** Faculty of Physics and Astronomy, Adam Mickiewicz University, Poznań 61-614, Poland

## Abstract

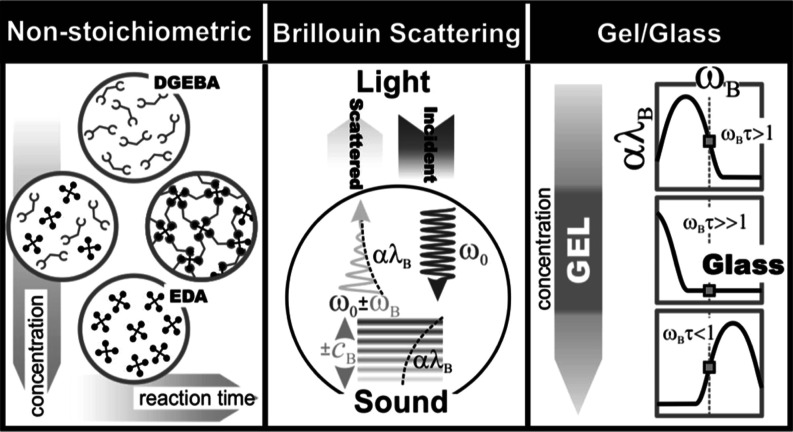

The initial and final stages of the isothermal curing reaction between diglycidyl ether of bisphenol A and ethylenediamine were investigated by using Brillouin light scattering spectroscopy. High-frequency acoustic parameters were measured for these stages as a function of the diamine molar fraction, ranging from the pure prepolymer to the pure cross-linker. Significant differences in the concentration dependencies of the mechanical parameters are interpreted in relation to changes in the system’s viscoelastic properties. At the final stage of the reaction, the concentration dependencies exhibit notable changes at two characteristic nonstoichiometric compositions—one corresponding to amine excess and the other to epoxy excess. While these concentrations align well with Flory’s critical gel points, experimental evidence suggests they should instead be interpreted as vitrification points.

## 1 Introduction

Epoxy resin is one of the most important thermosetting polymeric materials with excellent physicochemical properties, particularly desirable in many branches of industry. These are, for example, very good mechanical and electrical properties, mechanical and chemical stability under severe environmental conditions, and excellent adhesion to many different surfaces. Most of these advantageous properties appear when epoxy resin is cured to form an extended molecular entity (the gel and eventually, the glass) and depend mostly on the type and the relative amount of hardeners in the initial reacting system, physical conditions during curing, and the curing technology. In order to achieve desirable properties of cured epoxy resin as well as to search for new properties, many different hardeners are applied. These mainly belong to conventional small molecules, such as diamines,^1,2^ maleic anhydride,^3^ and methyl-tetrahydrophtacil anhydride.^4^ Recently, however, much bigger molecules, such as amino-terminated dendrimers,^5^ have been used as hardeners.

The mixtures of epoxy resin, such as, for example, digycidyl ether of bisphenol A (DGEBA), with aliphatic amines, besides purely applicable properties, have many advantages as a model compound in basic research. The advantage concerns quite simple reaction mechanisms which rely on stepwise polyaddition, where the polymerization process proceeds via the formation of chemical bonds at random between pairs of mutually reactive monomers.^6,7^ During such a reaction, molecular clusters are formed whose average size (average number of monomers per molecule) depends only on the functionality of the reagents and the number of chemical bonds formed. Moreover, the type of polymer formed during the reaction depends on the functionality of the amine; bifunctional amines yield linear chains, whereas reactions with multifunctional amines form a network polymer. The basic reaction taking place in these systems consists in the addition of amino hydrogen to the epoxy group, and because of the rigidity of DGEBA molecules, the formation of closed loop structures is negligible. All of these properties have been of primary importance in an interpretation of the results from the experiments applied in searching for the universal nature of the glass transition by observing the similarities between physical and chemical vitrification processes.^1,8−10^

Many different experimental techniques have been used to examine the properties of investigated epoxy systems, each of them only sensitive to specific physical properties. The most widely used are calorimetric methods, where the reaction enthalpy evolution is used in order to monitor the kinetics of the polymerization process. Among the others, the dielectric,^11−13^ fluorescence,^14^ rheometric,^12,15^ acoustical,^16,17^ or dynamic light scattering^9,10^ methods have been applied to examine particular properties of interest.

The experimental technique used in this study is the Brillouin light scattering (BLS) method, the light scattering method. The spectrum of the scattered light observed by this method arises mainly from local density fluctuation that propagates through the medium as a sound wave of gigahertz frequency, called a hypersound. Because of the direct coupling of hypersonic propagation properties (velocity and attenuation) with the mechanical properties of the investigated sample, the method is particularly useful in investigations of the dynamic and mechanical properties of the system at the hardly accessible GHz frequency range. Earlier studies of cross-linking systems have shown that this particular spectroscopic technique can be used as a very efficient and nondestructive method, particularly sensitive to the solidification of the sample.^15,18−26^

Most of the earlier works, with many different experimental techniques, dealt, however, only with stoichiometric systems. The variation of the stoichiometric ratio allows to examine a family of networks based on the same epoxy–amine pair. Such an approach provides a way of modifying the network structure without variation of the chemical nature of polymer chains and cross-links.^27,28^ In this study, we show the results of the high-frequency acoustical properties obtained for the mixtures of DGEBA and EDA with greatly unbalanced stoichiometry (covering the whole composition range), obtained at the beginning and after an isothermal curing of the system. The experimental results are analyzed with respect to the composition and reaction-induced changes in the structure and dynamics of nonstoichiometric systems, which result in alterations to the viscoelastic properties of the materials. These findings provide insights into the interplay between the coexisting and competing processes of gelation and vitrification across the full resin/hardener ratio range.

## 2 Experimental Section

The samples studied in this work consist of mixtures of DGEBA and ethylenediamine (EDA) as a hardener. DGEBA having the number-average molecular weight *M*_EP_ = 340 g/mol, epoxy equivalent γ_EP_ = 174 g/mol epoxy, and functionality *f*_EP_ = 2 has been supplied by Sigma-Aldrich. The analytical-grade pure EDA was supplied by Fluka. Its molecular weight was *M*_EDA_ = 60 g/mol and the functionality *f*_EDA_ = 4.

Samples were prepared that vary in initial amine molar fraction
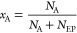
1where *N*_A_ and *N*_EP_ are the number of moles of diamine and epoxy resin, respectively. The molar ratios were chosen to prepare samples that cover a wide composition range: from an excess of epoxy groups to an excess of amino groups.

Before preparation of mixtures, pure DGEBA and EDA were first filtered using a 200 nm Millipore filter. Then, appropriate amounts of both substances were mixed together at temperature 293 K and stirred for about 5 min. The sample was then transferred into a glass vial (Daigger, USA) placed in a thermostatic holder, whose temperature was set to 293 K and controlled to the accuracy of ±0.1 K. The first light scattering experiment was performed just after sample preparation. The spectrum acquisition time was 180 s. The second experiment was performed after an isothermal curing of the mixture at 293 K. To make sure that the process had finished, the polymerization was carried out for 48 or 72 h depending on the initial mixture composition. The latter polymerization time was used for the systems where the diamine concentration, *x*_A_, was lower than 0.33.

During the Brillouin scattering experiment, the linearly polarized line (λ = 532 nm) of a Coherent DPSS 532 laser, working at a mean power of about 100 mW, was used as the probe. The VV component of scattered light was collected in a backscattering geometry and analyzed by a Sandercock-type (3 + 3)-pass Tandem Fabry–Perot interferometer, working at a free spectral range of 25 GHz with a finesse, estimated by the line width of the elastic line, of about 80.

In the kinetics experiment, the reaction proceeded for 12 h, and the BLS spectra were accumulated and recorded every 180 s.

**Figure 1:**
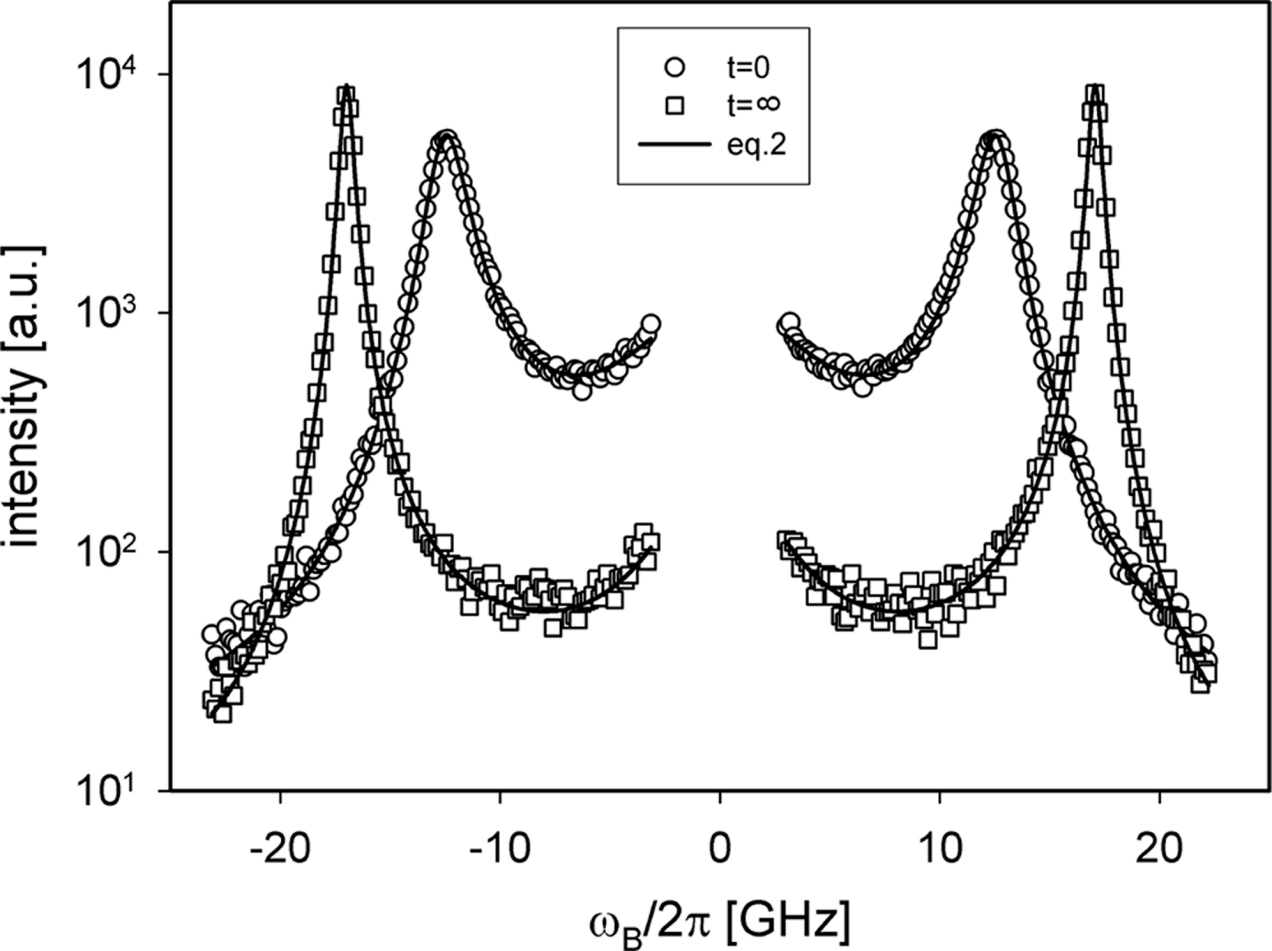
Semilog plot of the Brillouin spectra recorded at the initial (*t* = 0) and the final (*t* → ∞) stage of polymerization reaction for the sample with *x*_A_ = 0.33. Solid lines are the results of fitting with eq 2.

In Figure 1, we report the Brillouin spectra recorded at the initial and final stages of the polymerization reaction for the sample with an initial diamine composition *x*_A_ = 0.33. It is easily seen how the curing reaction influenced the shape of the phonon peaks. The lines recorded at the final stage of the reaction are shifted to higher frequencies and are much thinner compared to the ones registered at the beginning of curing. In addition, the presence of a central spectral component (the Mountain line) indicates the existence of high-frequency structural dynamics. In order to determine the values of the frequency shift and the line width, recorded spectra should be fitted to the appropriate model. We used the hydrodynamics expression for BLS spectrum,^29−32^ which is formally equivalent to the damped harmonic oscillator function with an additional central Mountain component^33^
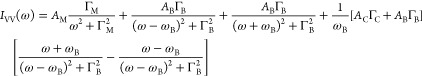
2

The first Lorentzian term of amplitude *A*_M_ describes the central Mountain line, whose half width at half maximum (HWHM), Γ_M_, is connected with rate of structural dynamics. The next two Lorentzians of amplitude *A*_B_ describe the symmetric contributions to the Brillouin scattering profile (Γ_B_ and ω_B_ are the HWHM and the frequency shift of the Brillouin line, respectively), whereas the last two terms represent the asymmetric contributions, arising from the first moment preservation selection rule. During fitting, the model function was convolved with the experimental resolution function, and free fitting parameters were *A*_M_, Γ_M_, *A*_B_, Γ_B_, and ω_B_.

The values of the parameters ω_B_ and Γ_B_, obtained by fitting eq 2 to experimental spectra registered at the initial (*t* = 0) and the final (*t* → ∞) stage of polymerization, correspond to the hypersonic wave velocity *v*_B_ and the attenuation of the hypersonic sound (α) per sound wavelength (λ_B_), αλ_B,_ through the following expressions^30,31,34^
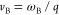
3a
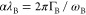
3bwhere *q* = (4π*n*/λ_0_)sin(θ/2) is the amplitude of the exchanged wave vector, λ_0_ is the incident wavelength, *n* is the refractive index of the medium, and θ is the scattering angle.

**Figure 2:**
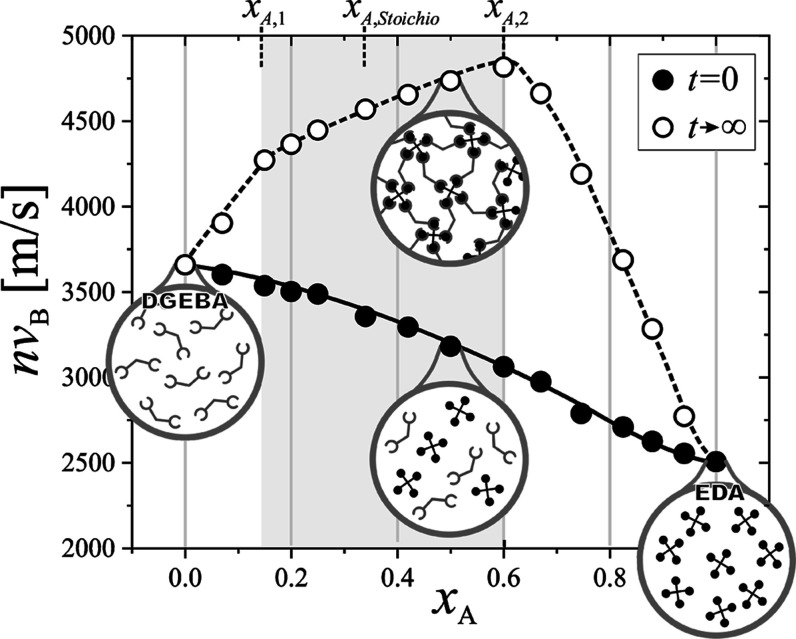
Concentration (amine molar fraction) dependence of the product of hypersound velocity and refractive index, *nv*_B_, registered at the initial (full symbols) and the final (open symbols) stages of polymerization. *x*_A,1_ and *x*_A,2_ are two critical gelation compositions. *x*_A,Stoichio_ corresponds to the stoichiometric composition. Lines are guides for the eye. Schematic representation of molecular structures is shown in insets. At the beginning of the reaction, the system is a homogeneous mixture. Formation of cross-links translates to the nontrivial mechanical behavior.

**Figure 3:**
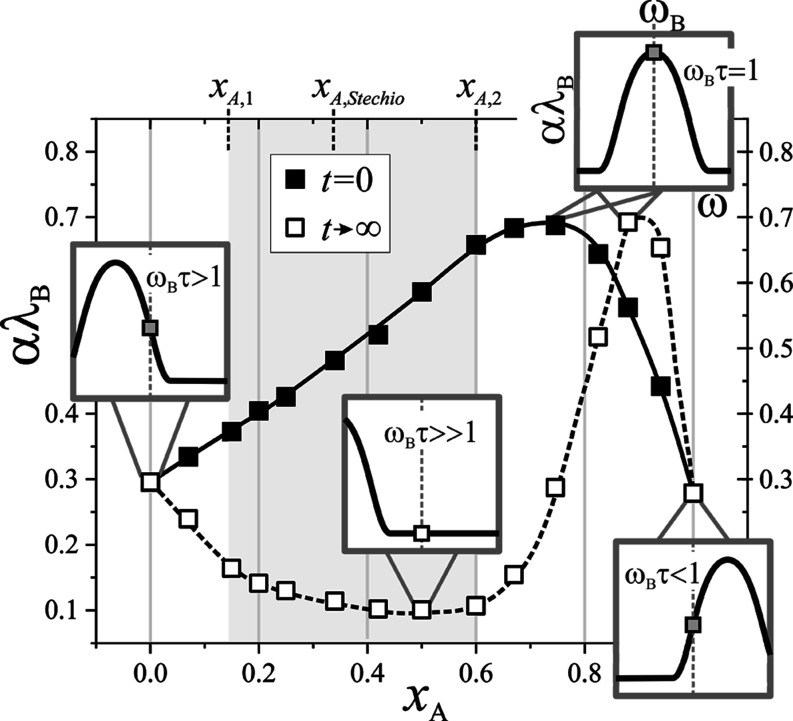
Concentration (amine molar fraction) dependence of hypersound attenuation, αλ_B_, registered at the initial (full symbols) and the final (open symbols) stages of polymerization. Lines are guides for the eye. Frequency dependencies of sound attenuation, αλ_B_(ω), for selected compositions are schematically illustrated within insets (scales are defined in the uppermost inset). The position of the curve, with respect to BLS frequency, ω_B_ (and so the value of αλ_B_ measured), depends on the actual dynamics (relaxation time, τ) of the system. It should be noted that ω_B_ is not strictly constant but also depends on composition and bond conversion.

The values of *n* for the thermosets studied are not known. For this reason, in the course of this paper, we are discussing dependencies of the product of hypersonic velocity *v*_B_ and refractive index *n*. The obtained values of *nv*_B_ and αλ_B_, as a function of epoxy molar fraction *x*_EP_, are displayed in Figures 2 and 3, respectively. It is worth noting that the total change in refractive index on curing of similar compounds at stoichiometric composition was less than 3%.^24^ The total change in hypersound frequency, Δω_B_, obtained for the currently investigated system at the same composition is about 40%. It is then justified to make further consideration of the *nv*_B_ product in terms of velocity alone.

## 3 Results and Discussion

Inspection of Figure 2 shows that, at the initial stage of reaction, the hypersound velocity is a monotonically decreasing function of the initial diamine molar fraction in the whole composition range. The dependence is almost linear with a small inflection toward lower velocity values observed for high amine concentrations (*x*_A_ = 0.7). At about the same composition, the dependence of the attenuation in the initial mixture (Figure 3) is characterized by a pronounced maximum. The dependencies of these parameters, as obtained when the curing reaction is finished, are distinctly different. The dependence of the velocity is no longer monotonic. The amine concentration evolution in this case can be divided into three distinct ranges. In the first one, 0<*x*_A_ ≤ *x*_A,1_, an increase in amine concentration causes the sound velocity in the final sample to increase, in opposition to the behavior observed for a nonreacted system. For intermediate initial amine concentrations, *x*_A,1_ < *x*_A_ ≤ *x*_A,2_, (shaded areas in Figures 2 and 3), the hypersonic speed of the cured system still increases. In this composition range, however, the slope of the dependence changes. Finally, for the highest cross-linker amount, *x*_A,2_ < *x*_A_ ≤ 1, the hypersound velocity decreases sharply to the value characteristic for neat amine. The αλ_B_(*x*_A_) obtained for polymerized samples seems to be connected to the corresponding velocity dependence. For diamine contents from the first composition range, the hypersonic attenuation decreases. In the second concentration range, the hypersound damping reaches a plateau-like minimum, and for even higher initial EDA contents (third concentration range), a pronounced peak appears, with the maximum located at *x*_A_ ≈ 0.9.

In order to interpret the above results, we have to find a theoretical formalism appropriate for the description of the experimental dependencies of the acoustic parameters. The linear viscoelasticity theory can be a suitable approach.^29,35^ In this framework, the dynamical structure factor, *S*(***q***, ω), describing the Brillouin scattering profile, depends on both the real and imaginary parts of the complex longitudinal modulus *M*(iω). In the theory of linear viscoelasticity, this parameter acts as a proportionality constant between longitudinal stress (introduced by acoustic pressure) and resulting deformation.

### 3.1 Viscoelastic Behavior

When the longitudinal wave is propagating through the sample, then the medium response to an alternating longitudinal stress can be characterized as a complex longitudinal modulus
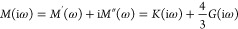
4where *G*(iω) is the complex rigidity modulus describing the answer of the medium to the pure shear stress, and *K*(iω) is a complex bulk modulus which corresponds to the purely compressional case.^34,35^ In some situations, an inverse of the longitudinal modulus, that is, adiabatic compressibility, β = 1/*M*′, is the parameter of importance.^36^

The velocity of the longitudinal wave, (*v*), and its absorption coefficient (α) per sound wavelength (λ_B_) can be then related to the real and imaginary parts of the longitudinal modulus, through the relations^34^
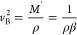
5
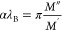
6

Both the complex shear *G*(iω) and the complex bulk *K*(iω) modulus can be divided into their real and imaginary parts describing ability to the accumulation and dissipation of the energy of appropriate mechanical deformation, respectively
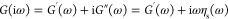
7
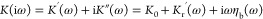
8where *K*r′(ω) and *K*_0_ are the relaxing parts of the compressional modulus and its zero-frequency value, respectively; η_s_(ω) is dynamical shear viscosity and η_b_(ω) is the dynamical bulk viscosity. The latter is formally related to any dissipation process other than shearing (i.e., chemical, conformational, or other processes). Using the above equations, the storage and the loss components of the complex longitudinal modulus, *M*(iω), can be expressed in the following form
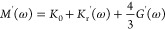
9
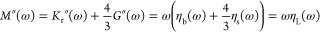
10where η_L_ is longitudinal viscosity, a composite property directly accessible in acoustical experiments^37,38^

After introduction of eqs 8 and 9 into relations 4 and 5, one gets the frequency dependencies for longitudinal sound velocity and the absorption coefficient
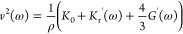
11
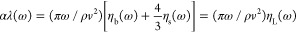
12where ρ is the density.

The above equations show that sound velocity can be taken as a probe of the rigidity of the system, whereas the value of the absorption coefficient depends on changes of the viscosity of the system. In order to interpret these two quantities, it is necessary to know the frequency dependencies of the appropriate parts of the moduli and viscosities. The simplest phenomenological model for stress relaxation in the case of the shape deformation of the liquid is the Maxwell model.^35^ According to this model, the complex longitudinal modulus depends on the product of the system relaxation time, τ, and the frequency of perturbation applied, ω
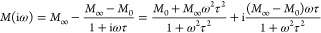
13

As follows from eq 12, the value of the storage modulus (real part) changes from *M*_0_ (relaxed value) for ωτ ≪ 1 to *M*_∞_ (unrelaxed value) for ωτ ≫ 1, whereas the loss modulus (imaginary part) vanishes in both limits. It is worth noting that the single relaxation model expressed by eq 12 does not take into account other potential processes (formally appearing in *G* and *K* moduli), proceeding at different time scales, which can account for some additional nonrelaxed residual loss.

Using eqs 9–12, the following relations for BLS measurables are obtained
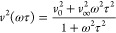
14
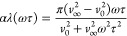
15
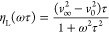
16where *v*_0_ and *v*_∞_ are, respectively, the relaxed and unrelaxed values of sound velocity.

Equations 13–15 indicate that the values of BLS measurables depend on both the limiting sound velocities and the product of frequency and relaxation time, ωτ. To accurately determine a constant relaxation time, experiments at multiple frequencies are required. In BLS, this is achieved by recording spectra at different scattering geometries.^32,39^ For current measurements conducted at a single scattering geometry and thus at frequencies limited to ω_B_, an alternative method is needed to vary ω_B_τ. This is most commonly accomplished by leveraging the strong temperature dependence of relaxation time.^40−42^ In the present isothermal case, changes in system dynamics can still be analyzed because the relaxation time is influenced by both the mixture concentration and the progress of the polymerization reaction, both of which affect the ω_B_τ product.

### 3.2 The Beginning of Cure

An almost linear dependence of *nv*_B_ suggests that the two reagents that form the investigated system are uniformly mixed in any proportion. Such behavior can be approximated by assuming ideal mixing of liquid molecules. Under these conditions, the compressibility β (∼*v*^–2^), of the binary liquid mixture is the volume-weighted sum of the pure liquids’ compressibilities.^36,43^ However, this description applies only to the relaxed values of sound velocities (or corresponding moduli and compressibilities), where no dynamic processes influence the sound velocities. This condition is not satisfied even for pure DGEBA, where the relaxed sound velocity (from ultrasonic measurements^23^) is approximately 2700 m/s, significantly lower than the ∼3600 m/s shown in Figure 2. As with the pure component, this dynamic process is also expected to manifest in unreacted mixtures. The presence of this dynamic behavior is more evident in sound absorption results.

As follows from eq 11, the value of the sound absorption coefficient depends on the viscosity of the system. In the case of a mixture of two components, its resultant viscosity depends on the values of viscosity coefficients for pure mixture components and their relative amount in the system. At investigated temperature (293 K), EDA is a low viscosity liquid, whereas DGEBA is in a supercooled state characterized by relatively high viscosity (the glass transition temperature for pure prepolymer, as obtained from differential scanning calorimetry, is 251 K^44^). Equation 15 relates the system’s viscosity coefficient to its structural relaxation time. In low-viscosity systems, structural reorganization occurs on a time scale much faster than that probed in typical Brillouin scattering experiments (tens of picoseconds). This corresponds to ω_B_τ<1 conditions. Contrarily, the structural relaxation process for supercooled DGEBA is well below the time scale window of the Brillouin spectroscopy method (ω_B_τ>1) and can be observed after increasing temperature to 350 K.^23,45^ When these two compounds are mixed together, one gets a system where the structural relaxation time (and so the viscosity) changes depending on the relative amount of both reagents. For high concentrations of DGEBA, the structural relaxation process is located at low frequencies (compared to the probe frequency of Brillouin spectroscopy), and our experimental method probes the high-frequency (or unrelaxed) value of sound velocity. Contrarily, for high diamine contents, the low-frequency (or relaxed) sound speed values are measured. The sound attenuation for these two limiting frequencies is low. When the cross-linker (EDA) is introduced to pure epoxy resin, the relaxation process of the initial mixture moves to higher frequencies, crossing the characteristic frequency of the Brillouin scattering method.

From the above consideration, the characteristic concentration for which the maximum in αλ_B_(*x*_A_) and inflection in *nv*_B_(*x*_A_) appears (*x*_A_ = 0.7 at Figure 3) corresponds to the situation, where the peak condition is met, i.e., when the probe frequency equals the relaxation frequency (ω_B_τ ≅ 1). For this condition, a transition from relaxed sound velocity, *v*_0_, toward the higher unrelaxed value, *v*_∞_, is also expected (eq 13), which can explain small inflection in *nv*_B_ at *x*_A_ = 0.7 (Figure 2).

### 3.3 Final Stage of Curing

After the reagents were mixed in specific proportions, the polymerization reaction (polyaddition) began. This reaction involves the formation of chemical bonds between mutually reactive monomers, leading to the creation of molecular clusters that grow in size and mass as the chemical conversion progresses. This process affects the system’s structural and dynamic properties. During polymerization, weak intermolecular interactions between the components of the mixture are replaced by strong covalent bonds between the corresponding functional groups. The number of possible bonds formed depends on the functionalities of the reagents and their concentrations. As the number of cross-links increases, the system becomes more rigid compared to the initial, nonbonded mixture, which is reflected in the increased value of the relaxed sound velocity *v*_0_.

If the number of chemical bonds formed between reacting molecules is high enough, then the initial molecular clusters, formed from interconnected reagent molecules (the sol phase), connect to form the network of bonds which spread out over the entire volume of the sample; the gel phase is formed. As gelation is approached, the static viscosity of the system increases dramatically, and the weight-average molecular weight tends to infinity. One could expect that gelation singularity would be somehow manifested also in the mechanical properties measured by BLS.

In addition to the stiffening effect, polymerization also influences the system’s dynamics. During the isothermal reaction, the growth of molecular clusters leads to a slowing down of molecular motion, reflected in the gradual increase in the structural relaxation time. This change in the system’s dynamics can be effectively described by Adam and Gibbs’ theory of cooperatively rearranging regions.^46^ According to this theory, the structural relaxation time is directly linked to the system’s configurational entropy. As the reaction proceeds, the formation of covalent bonds imposes the configurational restrictions on the system, causing the structural reorganization to become slower. Consequently, both *v*(ω) and αλ(ω) shift toward lower values on the ω scale (eq 13, insets in Figure 3). In this context, the pronounced maximum in sound attenuation observed for the reacted *x*_A_ = 0.9 sample (Figure 3) can be explained by the peak-like feature of the αλ(ωτ) function. For pure EDA measured at given ω_B_, ω_B_τ<1 is realized, resulting in low attenuation. As small amounts of resin are introduced, the system’s dynamics progressively slow down (relaxation time increases). At *x*_A_ = 0.9, the resonant condition (ω_B_τ = 1) is met, where attenuation reaches its maximum. Further addition of DGEBA increases the relaxation time of the cured system, leading to ω_B_τ>1, and as a result, attenuation decreases again. Changes in system dynamics will also impact the measured sound velocity. When ω_B_τ ≅ 1, there is a noticeable deviation from its relaxed value, *v*_0_ (which is already influenced by the formation of a network of rigid bonds), toward the higher unrelaxed value, *v*_∞_, as described by eq 13. As the chemical reaction proceeds, the increasing structural relaxation time also raises the system’s glass transition temperature, *T*_g_. If *T*_g_ reaches the constant polymerization temperature, the liquid’s dynamics become so slow (ω_B_τ ≫ 1) that the mixture vitrifies before the curing process is fully completed.^47^ Here, the influence of the main process responsible for sound attenuation vanishes, and low αλ_B_ values are recorded.

Both the gelation and vitrification processes depend on the initial proportions of the reacting mixture constituents. However, gelation occurs only within a limited concentration range, which can be estimated using the classical Flory–Stockmayer theory.^48^ For a curing reaction involving two compounds with functionalities of 2 and 4 (as in our case), gelation is predicted to occur within the 0.14 ≤ *x*_A_ ≤ 0.60 range.^21^ It is noteworthy that these values align closely with the *x*_A,1_ and *x*_A,2_ concentrations shown in Figure 2.

Although it may be tempting to attribute the changes in the slope of *nv*_B_(*x*_A_) for cured samples to the gelation process, caution is required when drawing such conclusions. This is because the curing process is not halted by gelation itself,^8−10^ provided the curing temperature is sufficiently high. Even in the gel state, the progressive slowing of system dynamics can eventually lead to the glass transition.

If the reaction were to reach full completion, the sound velocity (or rigidity) would be highest for the stoichiometric sample with *x*_A_ = 0.33, which corresponds to the sample with the highest possible density of cross-links. However, it is evident that this composition is not even distinguished in the *nv*_B_(*x*_A_) plot (Figure 2), suggesting that the cross-linking process was indeed interrupted before full completion. A similar conclusion can be drawn from the concentration dependence of the sound attenuation (Figure 3). In the *x*_A,1_ < *x*_A_ ≤ *x*_A,2_ range, αλ_B_ drops significantly and reaches very low values. This indicates that at 293 K, the curing reaction slows the structural relaxation time so drastically that most intermolecular motions are effectively frozen.

It is worth noting that even in this case, the Brillouin lines (Figure 1) were noticeably broader than the instrumental line of the spectrometer, and a central Mountain line was observed, indicating the presence of additional nonrelaxed processes that contribute to the energy dissipation of the hypersonic wave. In addition to intermolecular motions (associated with the phenyl rings of DGEBA), further damping arises from the motion of polymer chain segments that do not participate in network formation (dangling chains), as well as from unreacted mixture constituents trapped within the network structure, which contribute to network-fluid coupling.^49^

To confirm the glassy nature of a material, calorimetric measurements are typically performed. However, these tests require altering the sample’s temperature, which is unsuitable for systems driven to a glassy state through isothermal curing. In such cases, any increase in temperature would activate molecular motion of the reactants, further driving the polymerization and altering the system’s composition. Therefore, proper tests should be conducted under isothermal conditions, where the stage of the reaction is maintained.

**Figure 4:**
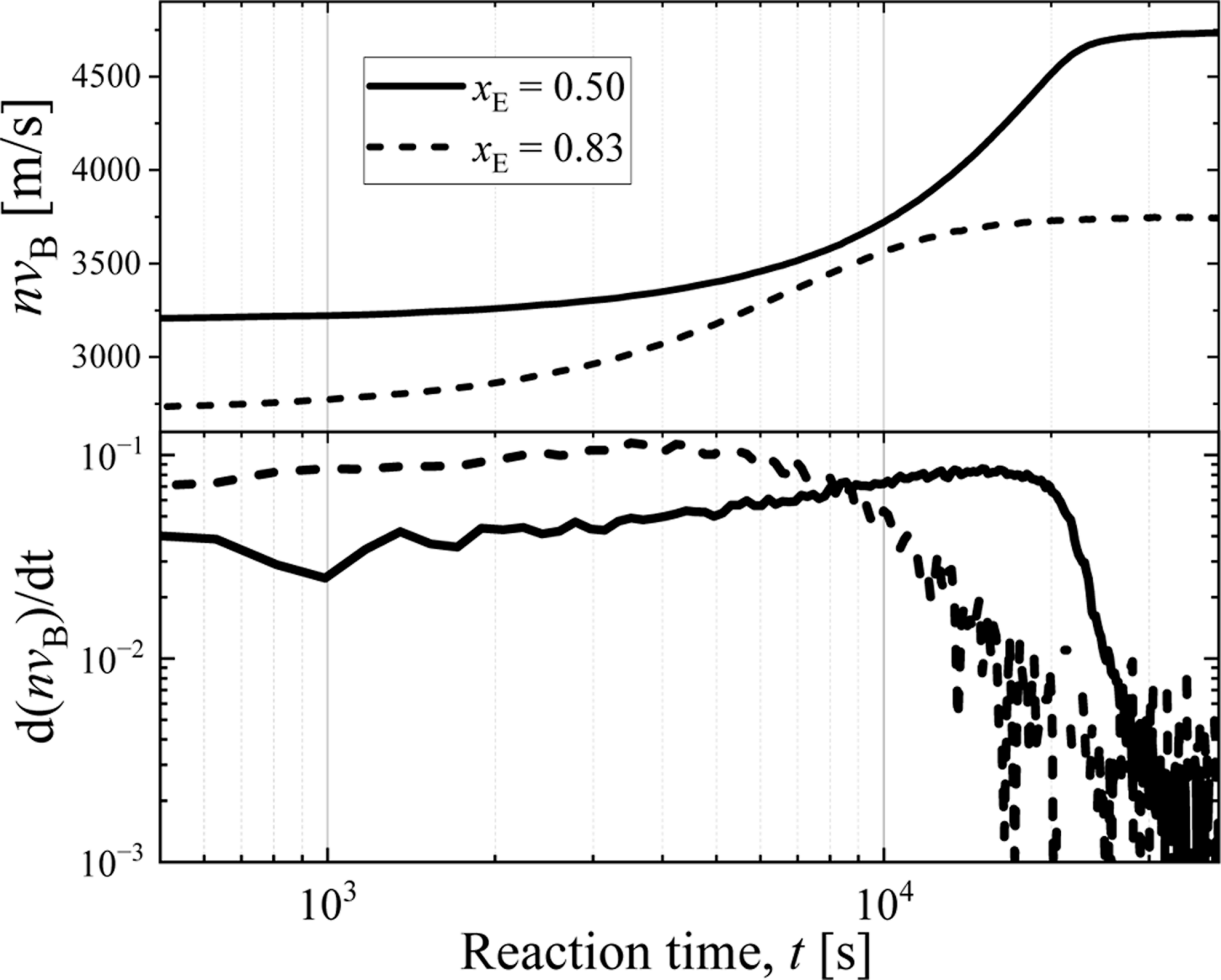
Hypersonic velocity (*nv*_B_—upper panel) and its time derivative (d*nv*_B_/d*t*) as a function of polymerization reaction time (log scale) for two mixtures being within or outside of *x*_A,1_ < *x*_A_ ≤ *x*_A,2_ gelation range (*x*_A_ = 0.50 and *x*_A_ = 0.83, respectively). Notice how the reaction stops drastically for the *x*_A_ = 0.5 system.

To verify the glassy nature of a moderately concentrated system, we measured the reaction kinetics at two compositions, *x*_A_ = 0.50 and *x*_A_ = 0.83, corresponding to values inside and outside the gelation range, respectively (Figure 4). The curing process proceeds smoothly and significantly faster in the initially less viscous mixture (*x*_A_ = 0.83). However, for the *x*_A_ = 0.50 system, the transition in the final stage of the reaction occurs abruptly, as expected for a dynamically arrested system.

The above considerations suggest that the distinct changes in the mechanical and dynamic properties of samples within the 0.14 ≤ *x*_A_ ≤ 0.60 range are associated with the glass transition. This conclusion is also supported by literature, which shows that even at relatively low epoxy conversions, the glass transition temperature for the stoichiometric DGEBA/EDA system is significantly higher than the reaction temperature of 293 K used in this study.^50,51^ Therefore, we conclude that the characteristic change in the slope of the *nv*_B_(*x*_A_) dependence is associated with dynamic arrest (glass transition) rather than gel transition phenomena. Notably, the velocity (or rigidity) of the system in this “glassy” range increases linearly with the initial amine molar fraction. This suggests that the density of cross-links is higher in amine-rich mixtures, where more bonds are formed before the reaction ceases, likely due to the lower viscosity of the initial EDA-rich mixtures.

## 4 Conclusions

We used the Brillouin spectroscopy technique to study the nonstoichiometric epoxy system based on DGEBA prepolymer cured isothermally with EDA. The samples were prepared to cover the whole composition range (from pure prepolymer to pure cross-linker), allowing us to obtain concentration dependence of the hypersonic wave velocity and attenuation at the initial and final stages of the curing process. Using the linear viscoelasticity theory, these acoustical parameters were connected with the sample rigidity and viscosity, allowing discussion of the change in the high-frequency mechanical properties arising from the isothermal curing process. The rigidity of the polymerized samples is always higher than initial mixtures, which is explained as a result of the formation of rigid covalent bonds in place of weaker interaction between molecules.

The composition dependence of the mechanical properties changes distinctly at two characteristic points, dividing the whole dependence into three regions. These distinctive compositions are in perfect agreement with the critical gel concentration values obtained by the Flory–Stockmayer theory. This result, however, is just a coincidence, and the composition region, which seems to be a gelation region, corresponds to the samples being vitrified. In this state, the rigidity of the medium increases linearly with the relative amount of the curing agent, suggesting that the glassy state for the amine excess system is characterized by a higher density of cross-links than in the epoxy excess situation.
